# Caveolin-3 KO disrupts t-tubule structure and decreases t-tubular *I*_Ca_ density in mouse ventricular myocytes

**DOI:** 10.1152/ajpheart.00209.2018

**Published:** 2018-07-20

**Authors:** Simon M. Bryant, Cherrie H. T. Kong, Judy J. Watson, Hanne C. Gadeberg, David M. Roth, Hemal H. Patel, Mark B. Cannell, Andrew F. James, Clive H. Orchard

**Affiliations:** ^1^School of Physiology, Pharmacology and Neuroscience, Biomedical Sciences Building, University of Bristol, Bristol, United Kingdom; ^2^Veterans Affairs San Diego Healthcare System and Department of Anesthesiology, University of California-San Diego, La Jolla, California

**Keywords:** calcium current, calcium release, calcium transient, caveolin-3

## Abstract

Caveolin-3 (Cav-3) is a protein that has been implicated in t-tubule formation and function in cardiac ventricular myocytes. In cardiac hypertrophy and failure, Cav-3 expression decreases, t-tubule structure is disrupted, and excitation-contraction coupling is impaired. However, the extent to which the decrease in Cav-3 expression underlies these changes is unclear. We therefore investigated the structure and function of myocytes isolated from the hearts of Cav-3 knockout (KO) mice. These mice showed cardiac dilatation and decreased ejection fraction in vivo compared with wild-type control mice. Isolated KO myocytes showed cellular hypertrophy, altered t-tubule structure, and decreased L-type Ca^2+^ channel current (*I*_Ca_) density. This decrease in density occurred predominantly in the t-tubules, with no change in total *I*_Ca_, and was therefore a consequence of the increase in membrane area. Cav-3 KO had no effect on L-type Ca^2+^ channel expression, and C3SD peptide, which mimics the scaffolding domain of Cav-3, had no effect on *I*_Ca_ in KO myocytes. However, inhibition of PKA using H-89 decreased *I*_Ca_ at the surface and t-tubule membranes in both KO and wild-type myocytes. Cav-3 KO had no significant effect on Na^+^/Ca^2+^ exchanger current or Ca^2+^ release. These data suggest that Cav-3 KO causes cellular hypertrophy, thereby decreasing t-tubular *I*_Ca_ density.

**NEW & NOTEWORTHY** Caveolin-3 (Cav-3) is a protein that inhibits hypertrophic pathways, has been implicated in the formation and function of cardiac t-tubules, and shows decreased expression in heart failure. This study demonstrates that Cav-3 knockout mice show cardiac dysfunction in vivo, while isolated ventricular myocytes show cellular hypertrophy, changes in t-tubule structure, and decreased t-tubular L-type Ca^2+^ current density, suggesting that decreased Cav-3 expression contributes to these changes in cardiac hypertrophy and failure.

## INTRODUCTION

Excitation-contraction coupling (ECC) in cardiac myocytes is initiated by the action potential, which activates sarcolemmal L-type Ca^2+^ channels (LTCCs), causing Ca^2+^ influx [Ca^2+^ current (*I*_Ca_)]. *I*_Ca_ triggers Ca^2+^ release from adjacent sarcoplasmic reticulum (SR) via Ca^2+^ release channels [ryanodine receptors (RyRs)]. This Ca^2+^-induced Ca^2+^ release (CICR) (20) produces local increases of cytosolic Ca^2+^ concentration [Ca^2+^ sparks (17)] that summate to form the cytosolic Ca^2+^ transient, leading to contraction. In ventricular myocytes, *I*_Ca_, and thus RyR activation, occurs predominantly at specialized invaginations of the sarcolemma called t-tubules (15, 34, 40). This arrangement achieves near-synchronous Ca^2+^ release (16), and thus contraction, throughout the cell. Relaxation occurs as cytosolic Ca^2+^ concentration decreases, mainly because of reuptake into the SR but also by removal from the cell via the Na^+^/ Ca^2+^ exchanger (NCX) (37).

Caveolin-3 (Cav-3) is a cholesterol-binding protein that is critical to the formation of caveolae and has been implicated in t-tubule formation (39) and localizing LTCC regulatory proteins and *I*_Ca_ to the t-tubules (2). C3SD peptide, which mimics the scaffolding domain of Cav-3 (19, 22), decreases *I*_Ca_ density at the t-tubules (36), which impairs local SR Ca^2+^ release (7, 11).

Recent work has revealed that cardiac hypertrophy and failure are accompanied by decreased Cav-3 expression and loss of Cav-3-dependent stimulation of *I*_Ca_ (11, 21, 23), and myocytes from failing hearts commonly display impaired SR Ca^2+^ release (8) as a result of altered regulation of LTCCs (11), decreased t-tubular *I*_Ca_ density (8, 9, 11), disruption of t-tubule organization (29, 45, 47), and reduced SR Ca^2+^ content (3, 24, 35).

These data suggest that decreased Cav-3 expression may underlie some of the phenotypic changes observed in heart failure (HF). Indeed, global genetic knockout (KO) of Cav-3 results in a progressive cardiomyopathy characterized by ventricular hypertrophy and dilation as well as reduced fractional shortening (48). Additionally, the loss-of-function mutation in Cav-3, T63S, has been associated with inherited hypertrophic cardiomyopathy (28). However, the extent to which decreased Cav-3 expression underlies the changes in ECC observed in HF is unknown. Therefore, we investigated the effect of Cav-3 KO on ECC in ventricular myocytes.

## METHODS

### 

#### Animals.

Adult (12 wk) male wild-type (WT) C57Bl/6 and homozygous Cav-3 KO mice, produced as previously described (27), were used. All animal procedures were approved by the University of Bristol local ethics committee and conducted in accordance with United Kingdom legislation [Animals (Scientific Procedures) Act 1986 Amendment Regulations 2012 incorporating European Directive 2010/63/EU].

#### Echocardiography.

In vivo cardiac structure and function were monitored using echocardiography. Animals were anesthetized (1–3% isoflurane), heart rate was monitored, and measurements of contractile performance were made from M-mode images acquired from the parasternal short axis view using a Vevo 3100 (FUJIFILM VisualSonics, Toronto, ON, Canada) and MX550D transducer.

#### Myocyte isolation and detubulation.

Ventricular myocytes were isolated using standard enzymatic digestion via Langendorff perfusion as previously described (7) and used on the day of isolation. Detubulation (DT), the physical and functional uncoupling of t-tubules from the surface membrane, was achieved using formamide-induced osmotic shock, as previously described (5, 6, 32).

#### Western blot analysis.

Protein samples [30 µg of heart homogenate for LTCCs, Cav-3, and junctophilin-2 (JPH-2); 25 µg of myocyte lysate for bridging integrator-1 (BIN-1)] were run on 4–15% gradient SDS-PAGE gels and transferred onto Immobilon-P membranes. Blots were probed with antibodies against Cav-3 (no. 610420, BD Transduction Laboratories, San Jose, CA, dilution 1:5,000), JPH-2 (no. 40-5300, ThermoFisher Scientific, Waltham, MA, dilution 1:500), BIN-1 (sc-23918, Santa Cruz Biotechnology, Dallas, TX, dilution 1:200), LTCCs (Alomone Laboratories, Jerusalem, Israel, dilution 1:1,000), and GAPDH (G9545, Sigma-Aldrich, St. Louis, MO, dilution 1:100,000). Protein bands were visualized and images were captured using horseradish peroxidase-conjugated secondary antibodies (W4011, α-rabbit horseradish peroxidase, Promega, Madison, WI, dilution 1:10,000; and W4021, α-mouse horseradish peroxidase, Promega, dilution 1:10,000), chemiluminescence, and a G:BOX Chemi XT4 imaging system (Syngene, Cambridge, UK). The density of the bands was measured using ImageJ (v1.50, National Institutes of Health, Bethesda, MD) and normalized to GAPDH.

#### Imaging and analysis of t-tubule structure.

Cell width and length were measured from bright-field images of isolated myocytes obtained using a 0.5 numerical aperture, ×16 oil-immersion objective; the large field of view enabled many cells to be captured in a single image. Cell volume was calculated from these measurements as previously described (4).

Surface and t-tubular cell membranes were labeled by incubating cells with 5 μmol/l di-8-ANEPPS (ThermoFisher) for 10 min. Image volumes were obtained using a LSM 880 confocal microscope (Zeiss, Carl Zeiss, Oberkochen, Germany) in Airyscan “superresolution” mode with a 1.2 numerical aperture, ×40 water-immersion objective, sampled at 40 nm in plane and 180 nm along the optical axis. The regularity of t-tubule staining was quantified by applying a two-dimensional (2-D) fast Fourier transform to an offset-subtracted square region of the interior of the cell, and the power of the first harmonic was normalized to that of the average image intensity (P_1_/P_0_). t-Tubule density and orientation were obtained by first processing the volumetric data with a tubule-enhancing three-dimensional (3-D) filter, segmenting using an Otsu threshold in MATLAB R2015a (Mathworks, Natick, MA), and converting to a skeleton using ImageJ. The skeleton was used to calculate t-tubule density [length per unit volume (in μm/μm^3^)] and local Eigenvectors for t-tubule angles. Tubule orientation is expressed relative to the z-disk plane, so that 0° corresponds to a transverse tubule and 90° corresponds to a tubule that is orthogonal to the z-disk, extending along the sarcomere (i.e., an “axial” tubule). Branch points were calculated using Skeletonize (2-D/3-D) in ImageJ.

DT efficiency was quantified by comparing t-tubule density in intact and DT cells labeled with di-8-ANEPPS, as previously described (8). In brief, one image from a volume stack was skeletonized, and t-tubule density was calculated as the length of tubule per cell area (in μm/μm^2^). DT efficiency (in %) was then defined as 1 – (DT cell t-tubule density/intact cell t-tubule density) × 100. DT efficiency was similar to that observed in previous studies and was not significantly different between cell types (WT: 90.1 ± 1.6%, *n* cells/*N* hearts: intact = 20/4 and DT = 15/2; and KO: 87.9 ± 2.3%; *n*/*N*: intact = 20/4 and DT = 24/3).

#### I_Ca_ recording.

Myocytes were placed in a chamber mounted on a Nikon Diaphot inverted microscope. Membrane currents and cell capacitance were recorded using the whole cell patch-clamp technique using an Axopatch 200B, Digidata 1322A analog-to-digital converter, and pClamp 10 (Molecular Devices, San Jose, CA). Pipette resistance was typically 1.5–3 MΩ when filled with pipette solution (see below), and pipette capacitance and series resistance were compensated by ~70%. Currents were activated from a holding potential of −80 mV by step depolarization to −40 mV for 200 ms (to inactivate Na^+^ current) followed by steps to potentials between −50 and +80 mV for 500 ms before repolarization to the holding potential, at a frequency of 0.2 Hz. Absolute *I*_Ca_ amplitude (in pA) in intact myocytes was measured as the difference between peak inward current and current at the end of the depolarizing pulse; absolute *I*_Ca_ in the t-tubular and surface membranes was calculated from measurements of *I*_Ca_ and membrane capacitance in intact and DT myocytes with correction for incomplete DT (DT efficiency) as previously described (8). *I*_Ca_ was normalized to cell capacitance (in pF; an index of membrane area) to calculate *I*_Ca_ density (in pA/pF). *I*_Ca_ density in the t-tubule membrane was calculated from the loss of membrane current and capacitance after DT; *I*_Ca_ density in the surface membrane was calculated from currents measured in DT myocytes with correction for incomplete DT as previously described (8).

#### NCX current recording.

To record NCX current (*I*_NCX_), BAPTA was omitted from the pipette solution (see below) and replaced with fluo-4 pentapotassium salt (ThermoFisher) to allow simultaneous measurement of intracellular Ca^2+^ and membrane current. Recordings were made 5 min after breaking into the cell to allow time for dialysis. Pipette resistance was typically 1.2–2 MΩ when filled with pipette solution. After a series of conditioning pulses (a 500-ms ramp from −80 to −40 mV followed by a step to 0 mV for 100 ms) at 1 Hz to steady state, stimulation was stopped and intracellular Ca^2+^ and *I*_NCX_ were recorded during application of 10 mM caffeine as previously described (26).

#### Latency and heterogeneity of SR Ca^2+^ release.

Intracellular Ca^2+^ and membrane potential were recorded simultaneously along single t-tubules in myocytes loaded with the Ca^2+^ indicator fluo-4/AM (5 μmol/l for 25 min, ThermoFisher) and the voltage-sensitive dye di-4-AN(F)EPPTEA [0.5–1 μg/ml for 15 min, kindly supplied by Dr. Leslie Loew (49)] as previously described (8). Cells were imaged using a Zeiss LSM 880 (see above) with the confocal pinhole set to 1 Airy unit. Line scans along a selected t-tubule were recorded at 0.51 ms/line, with excitation at 514 nm and emitted fluorescence collected between 518 and 560 nm for Ca^2+^ and 590–700 nm for voltage. Ca^2+^ release at the t-tubule was determined as described previously (8). In brief, di-4-AN(F)EPPTEA fluorescence was used to determine the upstroke of the action potential at the t-tubule. The latency of Ca^2+^ release at each point along the scan line was measured as the time between the upstroke of the action potential and the time when the Ca^2+^ signal became >5 SD above the average prestimulus value. Latency to time of maximum rate of rise of Ca^2+^ was also determined, and the SD of latencies for each cell was used as a measure of the heterogeneity of release. Line scans along the long axis of cells loaded with fluo-4/AM only were used to monitor spatially averaged whole cell Ca^2+^ transients. Cells were field stimulated at 0.2 Hz at 1.5× threshold using parallel Pt electrodes.

#### Solutions.

The standard superfusate for electrophysiology and imaging experiments contained (in mmol/l) 133 NaCl, 5 KCl, 1 MgSO_4_, 1 CaCl_2_, 1 Na_2_HPO_4_, 10 d-glucose and 10 HEPES, pH 7.4 (NaOH). During electrophysiological recordings, KCl was substituted with CsCl to inhibit K^+^ currents and the pipette solution contained (in mmol/l) 110 CsCl, 20 tetraethylammonium chloride, 0.5 MgCl_2_, 5 MgATP, 5 BAPTA, 10 HEPES, and 0.4 GTP-Tris pH 7.2 (CsOH). H-89 stock solution (10 mmol/l in distilled H_2_O) was diluted to 20 µmol/l in the superfusate for use. Cells were incubated in C3SD peptide (Pepceuticals Limited, Enderby, Leicestershire, UK, 1 µmol/l in 0.1 mmol/l Ca^2+^) for 1 h at room temperature before use (7, 36). All experiments were performed at room temperature.

#### Data presentation.

Data are expressed as means ± SE [of *N* animals for in vivo data and of *n* cells from *N* animals (*n*/*N*) for cellular measurements]. Data normality was assessed using the Shapiro-Wilk test, and subsequent testing was performed using a Student’s *t*-test or Mann-Whitney test, or one-way ANOVA or Kruskal-Wallis, as appropriate. *I*_Ca_ density-voltage relationship curves were analyzed using repeated-measures ANOVA with voltage and intervention (i.e. Cav-3 KO) as factors. Single myocyte properties, including those elicited by a step depolarization to a single voltage, were analyzed with two-way ANOVA; post hoc tests used Bonferroni correction. The errors in derived variables (specifically *I*_Ca_ density at the t-tubule membrane) and the subsequent statistical analysis (unpaired Student’s *t*-test) were calculated using propagation of errors from the source measurements. The limit of statistical confidence was taken as *P* < 0.05.

## RESULTS

### 

#### Cav-3 KO and cardiac function.

**Fig. 1.**
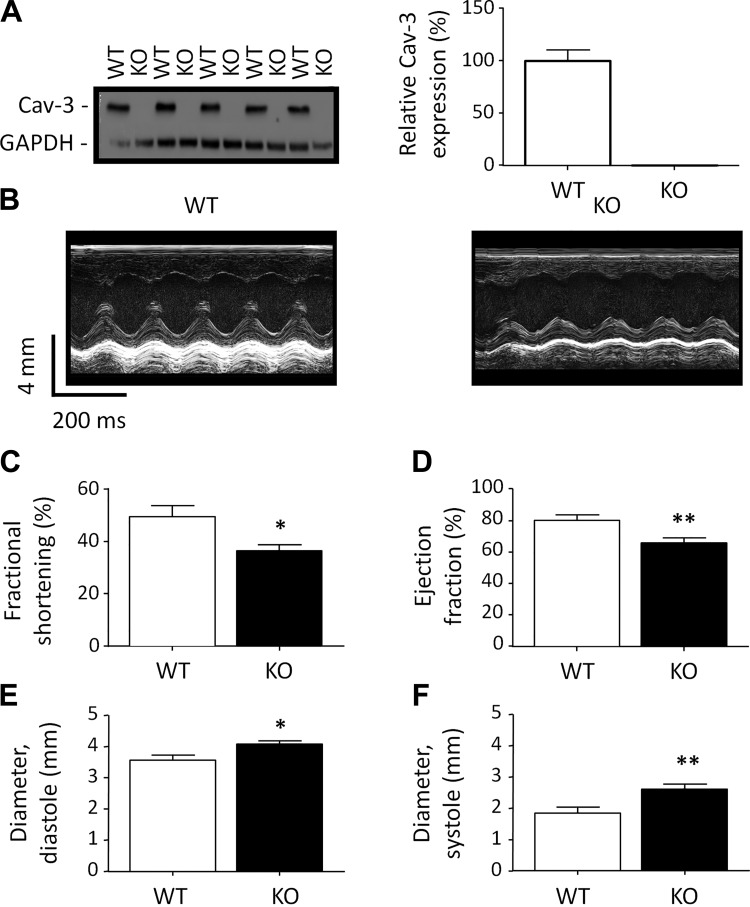
Caveolin-3 (Cav-3) and in vivo cardiac function. *A*: exemplar Western blots of Cav-3 (18 kDa) and GAPDH (37 kDa) in homogenates from wild-type (WT) and Cav-3 knockout (KO) mice (*left*) and mean densitometry data for Cav-3 Western blots (*N* = 5 animals in each group in duplicate; *right*). *B*: exemplar echocardiogram recordings from WT (*left*) and Cav-3 KO (*right*) mice. *C–F*: in vivo measurements of left ventricular cardiac function by echocardiography (WT: *N* = 6 and Cav-3 KO: *N* = 7). *C*: fractional shortening (in %). *D*: ejection fraction (in %). *E*: left ventricular internal diameter at diastole (in mm). *F*: left ventricular internal diameter at systole (in mm). *P < 0.05; **P < 0.01.

Western blots of heart homogenates for Cav-3 (Fig. 1*A*, *left*) and the associated densitometric analysis (Fig. 1*A*, *right*) confirmed that Cav-3 KO myocytes did not express detectable levels of Cav-3 protein compared with WT control myocytes.

Echocardiographic assessment of in vivo cardiac function showed a mild dilated cardiomyopathy, as reported previously (Fig. 1*B*) (48). WT and Cav-3 KO mice showed no difference in heart rate under anesthesia [WT: 436 ± 29 beats/min, *N* = 6, and Cav-3 KO: 437 ± 19 beats/min, *N* = 7, not significant (NS)]. Cav-3 KO was associated with a 26% decrease in fractional shortening (Fig. 1*C*) and an 18% decrease in ejection fraction (Fig. 1*D*), with significant increases in diastolic (Fig. 1*E*) and systolic (Fig. 1*F*) left ventricular internal diameters.

Despite the mild impairment of cardiac function, echocardiography showed no significant change in the mass (WT: 156.5 ± 21.0 mg and Cav-3 KO: 160.6 ± 14.4 mg, NS) or diastolic (WT: 1.1 ± 0.2 mm and Cav-3 KO: 1.0 ± 0.1 mm, NS) or systolic (WT: 1.6 ± 0.2 mm and Cav-3 KO: 1.5 ± 0.1 mm, NS) posterior wall thickness of the left ventricle. WT (*N* = 9) and Cav-3 KO (*N* = 8) mice also showed no significant differences in body weight (WT: 27.1 ± 0.7 g and Cav-3 KO: 27.8 ± 0.8 g, NS), tibia length (WT: 19.7 ± 0.3 mm and Cav-3 KO: 20.1 ± 0.3 mm, NS), heart weight-to-tibia length ratio (WT: 10 ± 0.5 mg/mm and Cav-3 KO: 9.4 ± 0.4 mg/mm, NS), or lung weight-to-tibia length ratio (WT: 8.3 ± 0.3 mg/mm and Cav-3 KO: 9 ± 0.7 mg/mm, NS). Thus, Cav-3 KO was associated with ventricular dilation but not cardiac hypertrophy or overt failure at 12 wk of age.

#### Ventricular myocyte morphology.

**Fig. 2.**
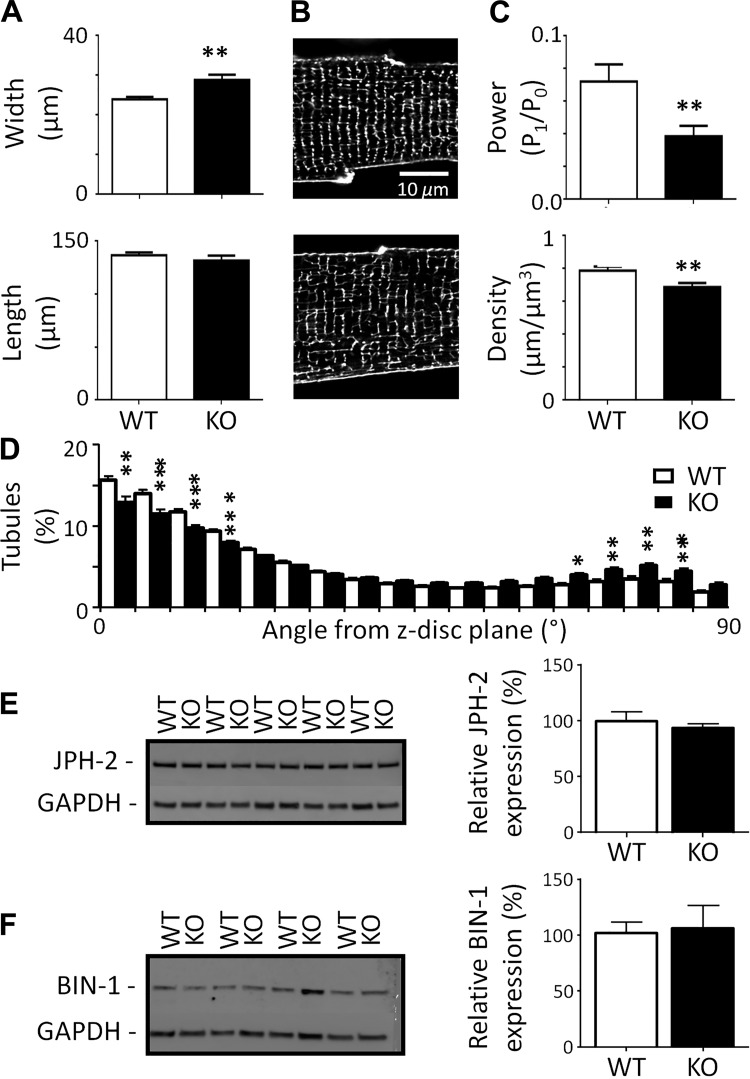
Morphology of isolated myocytes. *A*: cell width (*top*) and length (*bottom*) measured from bright-field images of myocytes from wild-type (WT) and caveolin-3 (Cav-3) knockout (KO) mice [WT *n* cells/*N* hearts (*n*/*N*): 100/4 and Cav-3 KO *n*/*N*: 30/4]. *B*: confocal images of t-tubules and surface sarcolemma stained with di-8-ANEPPs from representative WT (*top*) and Cav-3 KO (*bottom*) myocytes. *C*: t-tubule power (*top*) and density (*bottom*; WT *n*/*N*: 20/4 and Cav-3 KO *n*/*N*: 20/4). *D*: t-tubule orientation from the z-disk plane. *E* and *F*: Western blots of junctophilin-2 (JPH-2; 95 kDa; *E*) and bridging integrator-1 (BIN-1; 51 kDa; *F*) and GAPDH (37 kDa) (*left*) with corresponding mean data (*N* = 5 animals in each group in duplicate; *right*). *P < 0.05; **P < 0.01; ***P < 0.001.

Despite no evidence of hypertrophy at the organ level, myocytes isolated from Cav-3 KO hearts were increased in width (Fig. 2*A*) and thus calculated cell volume (WT: 47.0 ± 2.2 pl, *n*/*N* = 35/13, and Cav-3 KO: 58.8 ± 3.0 pl, *n*/*N* = 39/17, *P* < 0.01).

Staining the the surface membrane using di-8-ANEPPS revealed that t-tubule organization was also altered in Cav-3 KO myocytes. Figure 2*B* shows exemplar confocal images of WT (*top*) and Cav-3 KO (*bottom*) myocytes; the latter showed more prominent gaps in the network and an increase in the number of axial tubules. Quantification of t-tubule structure using 2-D fast Fourier transform analysis showed that t-tubule regularity, assessed as P_1_/P_0_ (see methods), was decreased in Cav-3 KO myocytes (Fig. 2*C*, *top*). More detailed 3-D analysis of image stacks revealed that this decrease was due to decreased t-tubule density (Fig. 2*C*, *bottom*) and changed tubule orientation (Fig. 2*D*); there was a 7% reduction in transversely oriented (0–15°) and an 8% increase in axially oriented (60–90°) tubules in Cav-3 KO myocytes. However, there was no significant difference in the number of branch points per length of tubule (WT: 0.48 ± 0.02 μm, *n*/*N* = 20/4, and Cav-3 KO: 0.45 ± 0.02 μm, *n*/*N* = 20/4, NS).

Since JPH-2 and BIN-1 have been implicated in determining t-tubule structure and localization of *I*_Ca_, we investigated whether Cav-3 KO altered expression of these proteins; however, JPH-2 and BIN-1 expression levels were not significantly different between WT and KO cardiac tissue (Fig. 2, *E* and *F*).

Thus, Cav-3 KO is associated with cellular hypertrophy and reduction in t-tubule density as well as changes in tubule orientation that occurred with no change in JPH-2 or BIN-1 expression.

#### Distribution of I_Ca_ and I_NCX_.

**Fig. 3.**
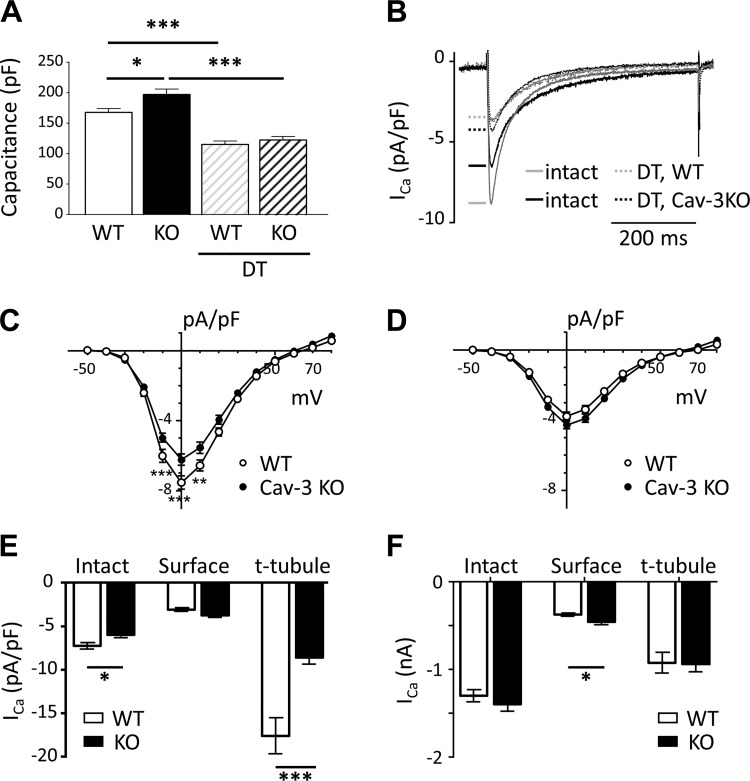
Membrane capacitance and Ca^2+^ current (*I*_Ca_). *A*: cell capacitance (in pF) of intact and detubulated (DT) myocytes used for electrophysiology from wild-type (WT) mice [intact *n* cells/*N* hearts (*n*/*N*): 35/13 and DT *n*/*N* = 38/13] and caveolin-3 (Cav-3) knockout (KO) mice (intact *n*/*N*: 39/17 and DT *n*/*N*: 43/14). *B*: exemplar records of *I*_Ca_ recorded at 0 mV from intact and DT WT and Cav-3 KO myocytes. *C* and *D*: mean *I*_Ca_ density-voltage relations from intact (*C*; WT *n*/*N* = 24/7 and Cav-3 KO *n*/*N* = 26/11) and DT (*D*; WT *n*/*N* = 28/9 and Cav-3 KO *n*/*N* = 31/9) WT and Cav-3 KO myocytes. *E* and *F*: mean *I*_Ca_ density (*E*) and absolute *I*_Ca_ (*F*) at 0 mV in intact and DT (“surface”) cells and calculated at the t-tubule membrane (“t-tubule”) for WT (open columns) and Cav-3 KO (solid columns) myocytes. *P < 0.05; **P < 0.01; ***P < 0.001.

Cells from Cav-3 KO mice showed a 17% increase in cell capacitance (a measure of membrane area; Fig. 3*A*), consistent with the 24% increase in calculated cell volume and 10% decrease of t-tubule density in these cells.

Figure 3*B* shows representative records of *I*_Ca_ elicited at 0 mV from intact and DT WT and Cav-3 KO myocytes; Fig. 3*C* shows the corresponding mean *I*_Ca_ density-voltage relationships for intact myocytes, demonstrating that Cav-3 KO was associated with reduced *I*_Ca_ density. This reduction in *I*_Ca_ density (WT: −7.53 ± 0.38 pA/pF, *n*/*N* = 24/7, and Cav-3 KO: −6.23 ± 0.33 pA/pF, *n*/*N* = 26/11, at 0 mV, *P* < 0.001; Fig. 3*E*) was due to the increase in cell capacitance because absolute *I*_Ca_ magnitudes were not significantly different in the two cell types (WT: −1,299 ± 70 pA, *n*/*N* = 24/7, and KO: −1,397 ± 81 pA, *n*/*N* = 26/11, at 0 mV, NS; Fig. 3*F*).

To examine the possible redistribution of *I*_Ca_ between surface and t-tubule membranes, some cells were detubulated before measurement of *I*_Ca_. DT had no significant effect on calculated cell volume but decreased cell capacitance in both WT and Cav-3 KO mice (Fig. 3*A*), consistent with loss of t-tubules, as previously described (5, 6, 32). Capacitance was not significantly different in DT WT and Cav-3 KO myocytes, suggesting that the increased capacitance observed in intact Cav-3 KO myocytes was predominantly due to an increase in t-tubule area.

DT significantly decreased *I*_Ca_ density in WT and Cav-3 KO myocytes to a level that was not significantly different in the two cell types (Fig. 3*D*), showing that *I*_Ca_ density at the surface membrane was not significantly different in Cav-3 KO and WT myocytes (Fig. 3*E*). However, absolute *I*_Ca_ was significantly higher in Cav-3 KO than WT myocytes (DT WT: −465 ± 25 pA, *n*/*N* = 28/9, and DT Cav-3 KO: −572 ± 33 pA, *n*/*N* = 31/9, at 0 mV, *P* < 0.05; Fig. 3*F*), so that despite a small (NS) increase in capacitance, *I*_Ca_ density was slightly (NS) higher at the surface of Cav-3 KO myocytes (Fig. 3, *D* and *E*). These data suggest that the significant decrease in *I*_Ca_ density observed in intact Cav-3 KO myocytes is due to a selective decrease in t-tubular *I*_Ca_ density. This was confirmed by calculation of t-tubular *I*_Ca_ density from these data, as previously described (8), which showed an ~51% decrease in t-tubular *I*_Ca_ density in Cav-3 KO compared with WT myocytes (Fig. 3*E*) with no change in absolute t-tubular *I*_Ca_ (Fig. 3*F*). Thus, the decrease of *I*_Ca_ density observed in Cav-3 KO ventricular myocytes is due to a decrease in t-tubular *I*_Ca_ density as the result of an increase in t-tubule area.

The scaffolding domain of Cav-3 has been suggested to bind directly to many signaling proteins, including adenylyl cyclase, thereby inhibiting their activity (19, 22, 46). To investigate whether loss of such inhibition might mask an underlying decrease in *I*_Ca_ in KO myocytes, we investigated the effect of C3SD peptide, which mimics the Cav-3 scaffolding domain, on *I*_Ca_ in Cav-3 KO myocytes. However, C3SD had no effect on *I*_Ca_ density (Cav-3 KO: −7.59 ± 0.48, pA/pF, *n*/*N* = 28/4, and Cav-3 KO plus C3SD: −7.96 ± 0.52 pA/pF, *n*/*N* = 28/4, at 0 mV, NS), although previous work has shown that tonic Cav-3-dependent stimulation of *I*_Ca_ is inhibited by C3SD in WT myocytes (11, 33). The lack of effect of C3SD on *I*_Ca_ in KO myocytes suggests that such stimulation is absent in these cells and thus other factors might be involved in maintaining absolute *I*_Ca_ in KO myocytes.

**Fig. 4.**
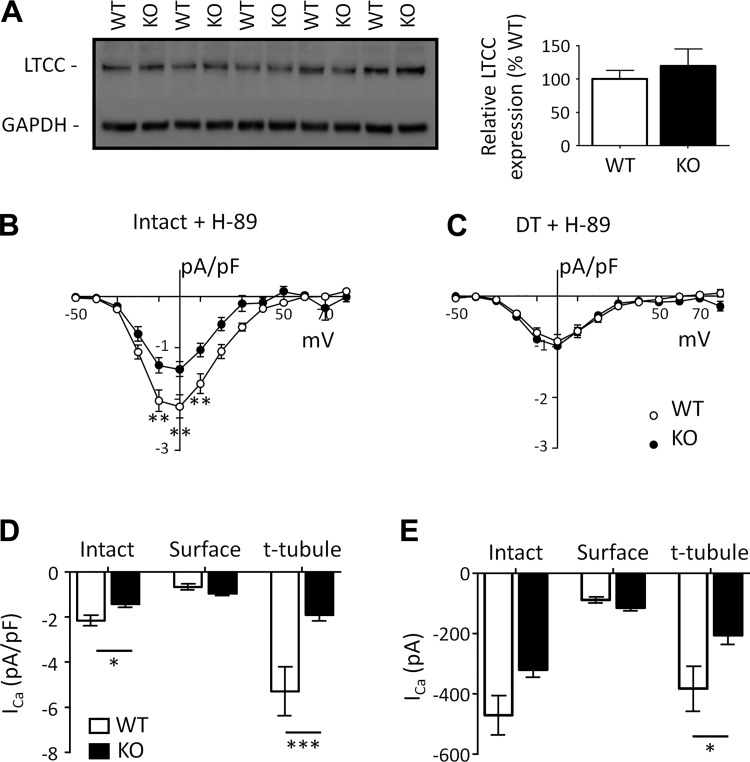
Ca^2+^ current (*I*_Ca_) in the presence of H-89. *A*: Western blots of the L-type Ca^2+^ channel (LTCC; 160 kDa) and GAPDH (37 kDa; *left*) and mean densitometry data (*N* = 5 animals in each group in duplicate; *right*). *B* and *C*: *I*_Ca_ density-voltage relations recorded in the presence of H-89 from intact [*B*; wild type (WT) *n* cells/*N* hearts (*n*/*N*): 16/9 and caveolin-3 (Cav-3) knockout (KO) *n*/*N*: 8/5] and detubulated (DT; *C*; WT *n*/*N*: 14/7 and Cav-3 KO *n*/*N*: 11/3) WT and Cav-3 KO myocytes. *D* and *E*: mean *I*_Ca_ density (*D*) and absolute *I*_Ca_ (*E*) at 0 mV in the presence of H-89 in intact and DT (“surface”) cells and calculated at the t-tubule membrane (“t-tubule”) for WT (open columns) and Cav-3 KO (solid columns) myocytes. *P < 0.05; **P < 0.01; ***P < 0.001.

We therefore investigated whether LTCC expression was increased in KO myocytes, which could help maintain *I*_Ca_. Figure 4*A* shows example Western blots for the LTCC α_1c_-subunit and mean data, which show that LTCC expression was unchanged in Cav-3 KO hearts compared with WT control hearts.

We also investigated whether *I*_Ca_ was maintained in KO myocytes as a result of increased PKA-dependent stimulation, since Cav-3 has been implicated in localizing and regulating constitutive PKA activity (41). Figure 4 shows the effect of the PKA inhibitor H-89 on mean *I*_Ca_ density-voltage relationships recorded from intact (Fig. 4*B*) and DT (Fig. 4*C*) myocytes from WT and Cav-3 KO mice. H-89 caused similar decreases in *I*_Ca_ density in intact (WT: 70% and Cav-3 KO: 77%, NS) and DT (WT: 70% and Cav-3 KO: 79%, NS) myocytes, so that *I*_Ca_ distribution was qualitatively similar in the absence and presence of H-89 in the two cells types (compare Figs. 3*E* and 4*D*). In the presence of H-89, absolute *I*_Ca_ was not significantly different in intact myocytes (WT: −471 ± 66 pA, *n*/*N* = 16/9, and Cav-3 KO: −321 ± 24 pA, *n*/*N* = 8/5, at 0 mV, NS); however, in contrast to the data obtained in the absence of H-89, and as shown in Fig. 4*E*, absolute *I*_Ca_ at the surface membrane was no longer significantly larger (WT: −127 ± 25 pA, *n*/*N* = 14/7, and Cav-3 KO: −140 ± 17 pA, *n*/*N* = 11/3, at 0 mV, NS) and absolute t-tubular *I*_Ca_ was now significantly smaller in KO myocytes (WT: −382 ± 74 pA, *n*/*N* = 54/16, and Cav-3 KO: −207 ± 29 pA, *n*/*N* = 32/8, at 0 mV, *P* < 0.05). These data suggest significant tonic stimulation of *I*_Ca_ by PKA at both the surface and t-tubule membranes in WT and KO myocytes, which is slightly greater in KO myocytes, helping to maintain absolute *I*_Ca_.

**Fig. 5.**
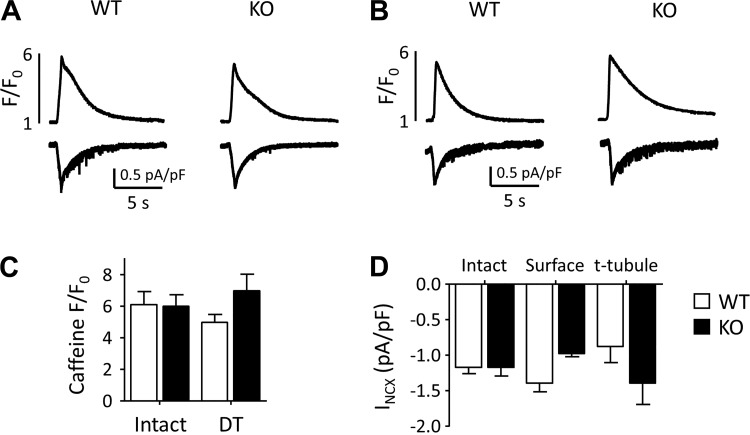
Intracellular Ca^2+^ and Na^+^/Ca^2+^ exchanger current (*I*_NCX_) during application of caffeine. *A* and *B*: exemplar records of the rise of intracellular Ca^2+^ (*top*) caused by application of 10 mM caffeine to intact (*A*) and detubulated (DT; *B*) wild-type (WT) and knockout (KO) myocytes and the accompanying inward currents (*I*_NCX_; *bottom*). *C*: mean amplitude of the caffeine-induced rise of intracellular Ca^2+^ [intact: WT *n* cells/*N* hearts (*n*/*N*): 11/6 and Cav-3 KO *n*/*N* = 13/6; DT: WT *n*/*N* = 10/4, Cav-3 KO *n*/*N* = 12/3]. *D*: distribution of *I*_NCX_ density, calculated as previously described (26).

Since Cav-3 KO decreased t-tubular *I*_Ca_ density, we also investigated the effect of Cav-3 KO on the main Ca^2+^ efflux pathway, *I*_NCX_. Figure 5*A* shows exemplar records of the rise of intracellular Ca^2+^ caused by application of 10 mM caffeine to intact WT and KO myocytes (*top*) and the accompanying inward currents (*I*_NCX_; *bottom*), showing no difference between the two cell types. Figure 5*B* shows corresponding traces obtained from DT WT and Cav-3 KO myocytes. Mean data showed that there was no difference in the amplitude of the caffeine-induced rise of intracellular Ca^2+^ in intact and DT WT and Cav-3 KO myocytes (Fig. 5*C*), suggesting no difference in SR Ca^2+^ content, and that *I*_NCX_ density, calculated as previously described (26), is similar at the surface and t-tubule membranes of WT myocytes, as previously described (26), and was not significantly different in WT and KO myocytes (Fig. 5*D*). However, the ratio of *I*_NCX_ at the cell surface to *I*_NCX_ at the t-tubules decreased from 1.59 ± 0.28 in WT myocytes to 0.70 ± 0.10 in KO myocytes (*P* < 0.01), suggesting a redistribution of *I*_NCX_ from the cell surface to the t-tubules after Cav-3 KO.

#### Intracellular Ca^2+^ handling.

**Fig. 6.**
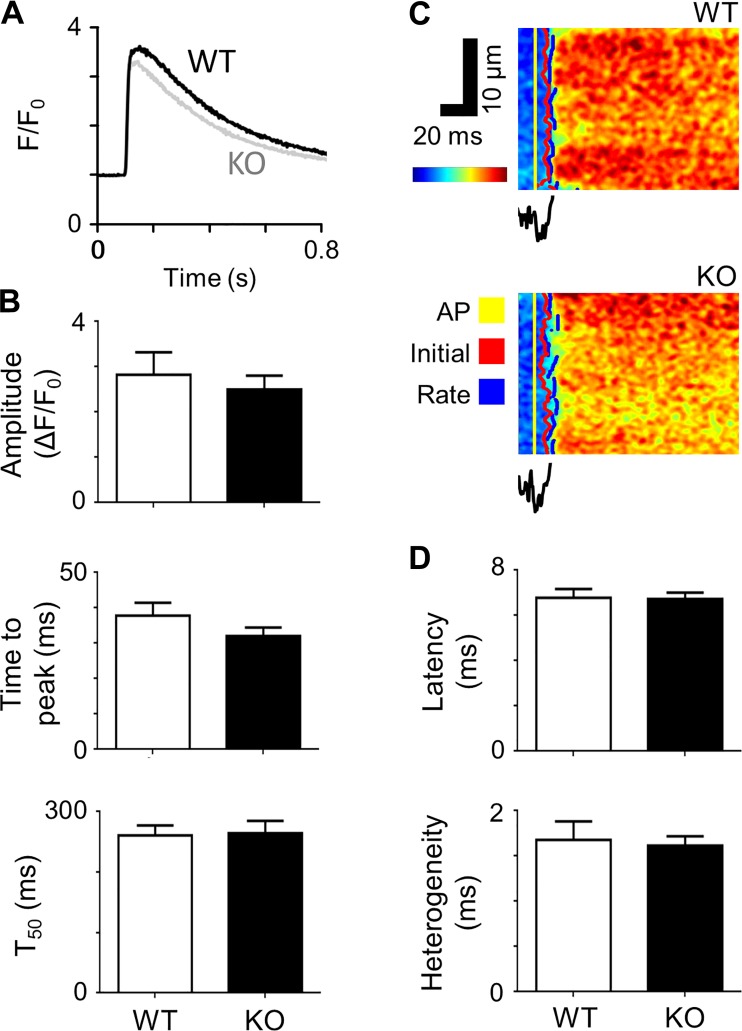
Systolic Ca^2+^ transients and local Ca^2+^ release. *A*: representative Ca^2+^ transients recorded from wild-type (WT) and caveolin-3 (Cav-3) knockout (KO) myocytes. *B*: Ca^2+^ transient amplitude, time to peak, and time to half decay (*T*_50_) measured from Ca^2+^ transients of WT [*n* cells/*N* hearts (*n*/*N*): 12/3] and Cav-3 KO (*n*/*N* = 14/3) cells. *C*: representative optical measurements of the rising phase of the Ca^2+^ transient and associated average t-tubular di-4-AN(F)EPPTEA signal. AP, action potential. *D*: mean latency and heterogeneity of sarcoplasmic reticulum Ca^2+^ release in WT (*n*/*N* = 10/3) and Cav-3 KO (*n*/*N* = 26/5) myocytes.

Since t-tubular *I*_Ca_ and SR Ca^2+^ content are key determinants of SR Ca^2+^ release, we investigated the effect of Cav-3 KO on Ca^2+^ release. Figure 6*A* shows representative systolic Ca^2+^ transients recorded from WT and Cav-3 KO myocytes, showing that Cav-3 KO had little effect on Ca^2+^ transient amplitude or time course. Mean data show that Cav-3 KO did not significantly change Ca^2+^ transient amplitude, time to peak, or time to half decay (Fig. 6*B*).

Closer examination also revealed little difference in local t-tubular CICR. Figure 6*C* shows representative line-scan images of fluo-4 fluorescence along a t-tubule with spatially averaged di-4-AN(F)EPPTEA fluorescence recorded from the t-tubule membrane in WT (*top*) and Cav-3 KO (*bottom*) myocytes. Measurement of the latency from the start of the action potential upstroke (yellow lines) to the initial Ca release along the t-tubule (red lines) revealed that Cav-3 KO had almost no effect (Fig. 6*D*). Similarly, neither the delay to the maximum rate of rise of Ca^2+^ (blue lines; mean data not shown) nor the variability in latency along the t-tubule (“heterogeneity”; Fig. 6*D*) were different in WT and Cav-3 KO myocytes.

## DISCUSSION

Previous work has shown that transverse aortic constriction (TAC)-induced hypertrophy and HF are associated with decreased cardiac Cav-3 expression (11). Although it has been suggested that Cav-3 inhibits hypertrophic signaling pathways (28, 48), the extent to which decreased expression underlies the cellular features of HF was unclear. The present study shows that Cav-3 KO causes mild dilated cardiomyopathy and cellular hypertrophy without overt HF. Cav-3 KO myocytes showed morphological and functional similarities to those from TAC-induced hypertrophic hearts (11), including cellular hypertrophy, disrupted t-tubule structure, and reduced t-tubular *I*_Ca_. Our data suggest that cellular hypertrophy underlies the accompanying decrease in density of t-tubules and t-tubular *I*_Ca_. Thus, decreased Cav-3 expression may underlie these changes in pathological conditions.

### 

#### In vivo and structural changes after Cav-3 KO.

The left ventricular dilatation and reduced fractional shortening and ejection fraction observed in vivo in Cav-3 KO mice confirm previous work using a different Cav-3 KO mouse line (48). These changes are unlikely to be due to changes in cardiac load, since blood pressure is unaltered after Cav-3 KO (48). They also occurred with no change in left ventricular mass, wall thickness, heart weight-to-tibia length ratio, or lung weight-to-tibia length ratio, and thus there is little evidence of cardiac hypertrophy or overt HF. Thus, since cellular hypertrophy was observed in isolated Cav-3 KO myocytes, loss of cell number must have occurred to maintain heart weight, consistent with the signs of cell death reported by Woodman et al. (48).

Cellular hypertrophy in Cav-3 KO myocytes was accompanied by an increase in cell capacitance, a decrease in t-tubule density, fewer transverse and more longitudinal tubules, and reduced t-tubule regularity. However, after DT, membrane capacitance was similar in WT and KO myocytes; this suggests that the increased capacitance in intact KO cells was due predominantly to increased t-tubule membrane area. The increase in capacitance is unlikely to be due to the inhibitory effect of Cav-3 KO on caveolae formation or cholesterol trafficking because *1*) it occurred predominantly at the t-tubules, *2*) loss of caveolae would decrease capacitance, and *3*) cholesterol depletion appears to have no effect on either t-tubule or surface membrane capacitance (25). We therefore used a previously published geometric model of ventricular cell morphology (33) to investigate the relationship between the observed changes in cell size and membrane areas. We incorporated the t-tubule membrane fraction determined from capacitance measurements in WT cells (31.2%) into the model and then simulated the increase in cell size and decrease in t-tubule density (11.3%) observed experimentally in KO myocytes. This resulted in a 12.2% increase in whole cell capacitance, consistent with that observed experimentally (17 ± 7%), and a t-tubular membrane fraction of 39.6%, which is very similar to that observed experimentally (37.6 ± 2.3%). This is the result of an increase in t-tubule area and fraction, predominantly caused by the increase in cell width (see Supplemental Figure S1 in Ref. 33), offset by the decrease in t-tubule density observed in KO myocytes. Thus, the changes observed in whole cell, surface membrane, and t-tubule capacitance, and thus fractional area, are consistent with, and can be explained by, the changes in cell geometry (cell size and t-tubule density) observed after Cav-3 KO.

The mechanism by which Cav-3 regulates cell and t-tubule growth is not clear. However, the present data showing that Cav-3 KO leads to cellular hypertrophy is consistent with the idea that Cav-3 inhibits the hypertrophic p42/44 MAPK pathway in the heart (48). The changes in the present study occurred with no change in JPH-2 or BIN-1 expression, suggesting *1*) that the decreased expression of these proteins observed in HF (e.g., Refs. 11 and 13) is not secondary to the associated decrease in Cav-3 expression and *2*) that the effects of Cav-3 KO cannot be ascribed to changes of JPH-2 and BIN-1 expression and thus that Cav-3 KO itself is responsible for the changes in t-tubule morphology. This may be the result of cellular hypertrophy without a commensurate increase in t-tubular growth or secondary to other changes, for example, mechanical changes (30) or changes in JPH-2 or BIN-1 distribution and/or function occurring as a result of Cav-3 KO.

These data suggest, therefore, that decreased Cav-3 expression contributes to the changes in t-tubule morphology observed in hypertrophy and HF. However, previous work from several laboratories has also implicated decreased expression of JPH-2 and BIN-1 in the altered t-tubule structure observed in HF (13, 24, 42, 43), and it is notable that the changes observed in the present study were smaller than those typically observed in HF, despite complete ablation of Cav-3 compared with the ~40% decrease reported in HF (11). Thus, other mechanisms, such as decreased JPH-2 and BIN-1 expression, must occur in parallel with the decrease in Cav-3 expression to account for the changes in t-tubule structure and ECC observed in HF.

In summary, Cav-3 KO causes cardiac dilatation, cellular hypertrophy, and changes in t-tubule organization; these cellular changes are similar to those observed in hypertrophic hearts but less marked than those in overt failure (11).

#### I_Ca_ and I_NCX_ in KO myocytes.

The present study shows that Cav-3 KO decreases t-tubular *I*_Ca_ density by increasing membrane area with no significant change in absolute *I*_Ca_, which is maintained, in part, by slightly greater PKA-dependent stimulation in Cav-3 KO myocytes. The decrease in density results in more uniform distribution of *I*_Ca_ between the t-tubule and surface membranes and was sufficient to cause a decrease in whole cell *I*_Ca_ density.

C3SD inhibits *I*_Ca_ in WT myocytes (11, 33) but had no effect on *I*_Ca_ in Cav-3 KO myocytes, which suggests, first, that the inhibitory effect of C3SD is mediated via interaction with Cav-3 rather than by a direct effect on the LTCC or its regulatory proteins. Recent studies have questioned the role of the Cav-3 scaffolding domain in binding to its protein partners (12, 18, 38); the observation that a peptide mimicking the scaffolding domain only inhibits *I*_Ca_ in the presence of Cav-3 suggests that the effect of Cav-3 on *I*_Ca_ is not due to the scaffolding domain binding alone but supports a role for the scaffolding domain in the physiological function of Cav-3. Second, it suggests that Cav-3-dependent stimulation of *I*_Ca_ is lost in Cav-3 KO myocytes and thus, third, that another, Cav-3-independent, mechanism contributes to the maintenance of *I*_Ca_ in Cav-3 KO myocytes. This Cav-3-independent mechanism is likely to involve PKA, because the inhibitory effect of H-89 on *I*_Ca_ appeared greater in KO than WT myocytes: the larger absolute *I*_Ca_ at the cell surface in Cav-3 KO myocytes in the absence of H-89 was no longer significant in the presence of H-89 and the similar absolute *I*_Ca_ at the t-tubules became significantly smaller in KO myocytes in the presence of H-89. Thus, although the distribution of *I*_Ca_ density was qualitatively similar in WT and Cav-3 KO myocytes in the absence and presence of H-89, it appeared to have a greater inhibitory effect on *I*_Ca_ in Cav-3 KO myocytes, consistent with increased stimulation of *I*_Ca_ by PKA in these cells (1). Interestingly, TAC-induced hypertrophy and HF are also associated with decreased t-tubular *I*_Ca_ density because of increased t-tubule surface area with no significant change in absolute *I*_Ca_ and with loss of response of *I*_Ca_ to C3SD (11). This suggests that Cav-3-dependent stimulation of *I*_Ca_ is absent and thus absolute *I*_Ca_ is maintained in both Cav-3 KO and after TAC by other factor(s), which appear to include increased PKA activity (the present study and Refs. 31 and 44). This also suggests that decreased Cav-3 expression may, in part, underlie these changes in hypertrophy and failure; although it remains possible that the changes observed after Cav-3 KO are secondary to remodeling, such changes would also, presumably, occur because of decreased Cav-3 expression under pathological conditions.

Although the present data are consistent with previous work showing PKA-dependent stimulation of *I*_Ca_ at the t-tubule and surface membranes of mouse myocytes (33), it is unclear why Cav-3 KO increases PKA-dependent stimulation of *I*_Ca_ at the t-tubular and surface membranes, since Cav-3 colocalizes with LTCCs and components of the PKA cascade in a signaling domain at t-tubules (2). Furthermore, previous work has shown that acute treatment of cells with C3SD peptide inhibits local Cav-3-dependent stimulation of a subset of LTCCs by PKA, probably by disrupting the signaling domain (2, 7, 9). Presumably, chronic ablation of Cav-3 disrupts the localization of PKA activity to signaling domains, resulting in the stimulation of more LTCCs.

The reason for the smaller absolute t-tubular *I*_Ca_ in KO myocytes in the presence of H-89 is also unclear since expression of LTCCs and BIN-1 (which has been implicated in trafficking LTCCs to the t-tubules) was unchanged. It could be due to loss of Cav-3-dependent stimulation of *I*_Ca_ (above) without the compensatory increase in stimulation by PKA but may reflect relocation of LTCCs. Together, however, the data show that the decrease in t-tubule, and thus whole cell, *I*_Ca_ density in Cav-3 KO myocytes is due to the increase in t-tubule area, partly offset by increased stimulation by PKA.

In contrast to *I*_Ca_, Cav-3 KO did not alter *I*_NCX_ density in the intact cell, nor did it significantly alter *I*_NCX_ at surface and t-tubule membranes. However, Cav-3 KO significantly decreased the ratio of *I*_NCX_ at the cell surface to *I*_NCX_ at the t-tubules, suggesting redistribution of *I*_NCX_ from the cell surface to the t-tubular membrane. This redistribution is the reverse of that observed for *I*_Ca,_ so that Cav-3 appears to have reciprocal effects on the distributions of *I*_Ca_ and *I*_NCX_, although the mechanism of regulation of *I*_NCX_ distribution is unknown. However, the lack of effect of Cav-3 KO on *I*_NCX_ density in the intact cell implies some upregulation of *I*_NCX_ so that its density remains unchanged despite the cellular hypertrophy induced by Cav-3 KO.

#### SR Ca^2+^ release.

Although *I*_Ca_ density was reduced in Cav-3 KO myocytes, particularly at the t-tubules, neither the amplitude nor time course of the whole cell Ca^2+^ transient amplitude was altered. More detailed examination of CICR by measuring the latency and heterogeneity of local Ca^2+^ release along a t-tubule also revealed no effect of Cav-3 KO.

Ca^2+^ release was maintained with no significant change in SR Ca^2+^ content and may be explained by redundancy in t-tubular *I*_Ca_ in ventricular myocytes (14). However, it is also possible that *1*) dyadic *I*_Ca_ was maintained, since absolute *I*_Ca_ was unchanged in KO myocytes; *2*) a larger population of LTCCs is stimulated by PKA in KO myocytes (above), helping to maintain CICR; *3*) Ca^2+^/calmodulin-dependent protein kinase, which was inhibited by BAPTA for the measurement of *I*_Ca_, helps maintain *I*_Ca_ in intact myocytes; and *4*) RyR sensitivity increased because of increased PKA-dependent phosphorylation.

However, the lack of effect of Cav-3 KO on local Ca^2+^ release compared with the increased latency and heterogeneity of Ca^2+^ release at the t-tubule observed in HF suggests that other factors may be affecting Ca^2+^ release in HF. One possibility is dyadic disruption in HF as a result of decreased expression of JPH-2 and BIN-1, which have been implicated in dyad formation (13, 24, 42, 43), whereas expression of these proteins was unchanged in KO myocytes. Similarly, the relatively small disruption of t-tubule structure compared with HF might explain why there was relatively little effect on the systolic Ca^2+^ transient. Thus, the reduction in *I*_Ca_ density and disruption of t-tubule organization associated with Cav-3 KO were insufficient to cause significant changes of Ca^2+^ release, in contrast to those observed in HF.

These data also suggest that the decrease in ejection fraction observed in Cav-3 KO mice in vivo is not due to altered Ca^2+^ release, although it remains possible that Ca^2+^ transient amplitude may be altered by Cav-3 KO at higher (physiological) frequencies. However, the decrease occurred in the presence of increased heart size; since wall tension will increase with dilation, the law of Laplace shows that a reduced ejection fraction does not necessarily imply reduced contractility. Impaired cardiac function in KO mice is also associated with an increase in extracellular matrix and fibrosis (48), which, with possible changes in neurohumoral influences in vivo, may contribute to the decrease in ejection fraction.

#### Conclusions.

These data show that decreased Cav-3 expression is likely to contribute to the cellular hypertrophy, disrupted t-tubule structure, and decreased *I*_Ca_ density observed in hypertrophy but not with the more marked changes observed in HF.

## GRANTS

This work was funded by British Heart Foundation Grants PG/14/65/31055 (to C. H. Orchard and A. F. James) and RG/12/10/29802 (to C. H. Orchard, A. F. James, and M. B. Cannell); National Institutes of Health HL091071 (to H. H. Patel), HL066941 (to H. H. Patel and D. M. Roth), andAG052722 (to H. H. Patel); and Veterans Affairs Merit BX001963 (to H. H. Patel) and BX000783 (to D. M. Roth).

## DISCLOSURES

No conflicts of interest, financial or otherwise, are declared by the authors.

## AUTHOR CONTRIBUTIONS

S.M.B., C.H.T.K., J.J.W., H.C.G., D.M.R., H.H.P., M.B.C., A.F.J., and C.H.O. conceived and designed research; S.M.B., C.H.T.K., J.J.W., and H.C.G. performed experiments; S.M.B., C.H.T.K., J.J.W., and H.C.G. analyzed data; S.M.B., C.H.T.K., J.J.W., H.C.G., A.F.J., and C.H.O. interpreted results of experiments; S.M.B., C.H.T.K., and H.C.G. prepared figures; S.M.B., C.H.T.K., and C.H.O. drafted manuscript; D.M.R., H.H.P., M.B.C., and A.F.J. edited and revised manuscript; C.H.O. approved final version of manuscript.
